# Norfloxacin-Loaded Electrospun Scaffolds: Montmorillonite Nanocomposite vs. Free Drug

**DOI:** 10.3390/pharmaceutics12040325

**Published:** 2020-04-04

**Authors:** Angela Faccendini, Marco Ruggeri, Dalila Miele, Silvia Rossi, Maria Cristina Bonferoni, Carola Aguzzi, Pietro Grisoli, Cesar Viseras, Barbara Vigani, Giuseppina Sandri, Franca Ferrari

**Affiliations:** 1 Department of Drug Sciences, University of Pavia, Viale Taramelli 12, 27100 Pavia, Italy; angela.faccendini@gmail.com (A.F.); marco.ruggeri02@universitadipavia.it (M.R.); dalila.miele@gmail.com (D.M.); silvia.rossi@unipv.it (S.R.); cbonferoni@unipv.it (M.C.B.); pietro.grisoli@unipv.it (P.G.); barbara.vigani@unipv.it (B.V.); franca.ferrari@unipv.it (F.F.); 2 Department of Pharmacy and Pharmaceutical Technology, Faculty of Pharmacy, University of Granada, Campus of Cartuja, 18071 Granada, Spain; carola@ugr.es (C.A.); cviseras@ugr.es (C.V.)

**Keywords:** electrospinning, chitosan, glycosaminoglycans, scaffolds, fibroblasts proliferation, antimicrobial properties

## Abstract

Infections in nonhealing wounds remain one of the major challenges. Recently, nanomedicine approach seems a valid option to overcome the antibiotic resistance mechanisms. The aim of this study was the development of three types of polysaccharide-based scaffolds (chitosan-based (CH), chitosan/chondroitin sulfate-based (CH/CS), chitosan/hyaluronic acid-based (CH/HA)), as dermal substitutes, to be loaded with norfloxacin, intended for the treatment of infected wounds. The scaffolds have been loaded with norfloxacin as a free drug (N scaffolds) or in montmorillonite nanocomposite (H—hybrid-scaffolds). Chitosan/glycosaminoglycan (chondroitin sulfate or hyaluronic acid) scaffolds were prepared by means of electrospinning with a simple, one-step process. The scaffolds were characterized by 500 nm diameter fibers with homogeneous structures when norfloxacin was loaded as a free drug. On the contrary, the presence of nanocomposite caused a certain degree of surface roughness, with fibers having 1000 nm diameters. The presence of norfloxacin–montmorillonite nanocomposite (1%) caused higher deformability (90–120%) and lower elasticity (5–10 mN/cm^2^), decreasing the mechanical resistance of the systems. All the scaffolds were proven to be degraded via lysozyme (this should ensure scaffold resorption) and this sustained the drug release (from 50% to 100% in 3 days, depending on system composition), especially when the drug was loaded in the scaffolds as a nanocomposite. Moreover, the scaffolds were able to decrease the bioburden at least 100-fold, proving that drug loading in the scaffolds did not impair the antimicrobial activity of norfloxacin. Chondroitin sulfate and montmorillonite in the scaffolds are proven to possess a synergic performance, enhancing the fibroblast proliferation without impairing norfloxacin’s antimicrobial properties. The scaffold based on chondroitin sulfate, containing 1% norfloxacin in the nanocomposite, demonstrated adequate stiffness to sustain fibroblast proliferation and the capability to sustain antimicrobial properties to prevent/treat nonhealing wound infection during the healing process.

## 1. Introduction

The skin is the major protective barrier against the environment and the loss its integrity, as a result of injury or illness, may lead to morbidity or even death.

Wound healing is a complex event, based on overlapping but well-orchestrated cellular and molecular processes, to repair damaged tissue and restore skin function [1,2]. The process of healing proceeds through different phases (hemostasis, inflammatory, proliferative and remodeling) and involves extracellular matrix (ECM) molecules, soluble mediators, as cytokines and growth factors, various resident cells, and infiltrating leucocytes. In nonhealing wounds, the healing process stops at the inflammatory state, and chronic wounds, such as venous leg ulcers, arterial ulcers, diabetic ulcers, and pressure ulcers, i.e., bed sores, fail to proceed through an orderly and timely process to restore skin anatomical and functional integrity [1,2]. Moreover, all of these wounds are contaminated by proliferating bacteria from the surrounding skin, the local environment, and the endogenous patient sources, resulting in wound colonization [3,4]. This could enhance or impair wound healing, depending on the bacterial load. In the absence of an effective immune response, impeded by underlying morbidity, as venous and arterial insufficiency, diabetes, or ageing, bacterial colonization becomes critical and an unavoidable transition towards infection occurs [3,4]. In fact, the exposed subcutaneous tissue provides a favorable substrate for the microbial growth of a wide variety of microorganisms. Moreover, a longer healing time could dramatically increase the possible occurrence of infection and biofilm formation [4,5].

Infections in nonhealing wounds remain one of the major challenges. Although appropriate systemic antibiotics are considered essential for the treatment of clinically infected wounds, topical antibiotics are not recommended since they could promote bacterial resistance. Recently, a nanomedicine approach, creating antimicrobial nanotherapeutics, has appeared to be a valid option to eliminate bacterial infections, since nanomaterials can overcome antibiotic resistance mechanisms, owing to their unique and advantageous physico-chemical properties [6,7]. In fact, several studies report that nanosystems interact with microorganisms upon multiple mechanisms, including electrostatic attraction, hydrophobic and Van der Waals forces through surface interactions, and this makes them promising candidates to achieve enhanced therapeutic efficacy against multidrug resistant (MDR) infections [6,7]. Considering this evidence, in this work, a norfloxacin–montmorillonite nanocomposite (VHS-N), previously prepared by an intercalation solution procedure, was encapsulated in nanofibrous scaffolds, since it proved to increase drug potency against both *Pseudomonas aeruginosa* and *Staphylococcus aureus* (probably due to the high surface area to volume ratio, which increases the contact area with target organisms), maintaining cytocompatibility towards fibroblasts in vitro [8].

Given this premise, the aim of this study was the loading of montmorillonite norfloxacin nanocomposite (VHS-N) in three types of biopolymer–polysaccharide-based scaffolds (chitosan-based (CH), chitosan/chondroitin sulfate-based (CH/CS), chitosan/hyaluronic acid-based (CH/HA) (H hybrid scaffolds) to obtain dermal substitutes, intended for the treatment of wounds prone to infection, such as chronic ulcers (diabetic foot, venous leg ulcers) and burns.

The hybrid scaffolds were compared with scaffolds with the same compositions in polysaccharides, but loaded with norfloxacin as a free drug (N scaffolds).

The unloaded scaffolds were previously designed and developed [9,10]. Briefly, chitosan and chitosan/glycosaminoglycan electrospun scaffolds were manufactured using electrospinning by means of a simple/single-step process. Polymeric blends in water/acetic acid mixture were electrospun and the resulting random scaffolds were crosslinked by heating to obtain water resistant systems. The scaffolds proved their effectiveness in enhancing cell growth in vitro (fibroblasts and endothelial cells) and wound healing in vivo in a murine, burn/excisional model [9]. Moreover, lysozyme, normally secreted by macrophages and polymorphonuclear neutrophilis during the inflammatory phase of the healing process, proved to degrade the scaffolds in vitro [10].

Chitosan, glycosaminoglycans and pullulan were selected since they are polysaccharide biopolymers (organic molecules synthesized by the living organisms [11]), and biopolymers are recognized as the most promising materials in wound healing since they are characterized by having many advantages over synthetic materials because of their biocompatibility, biodegradability, lower antigenicity and renewability [11]. Therefore, although there are some examples in the literature focused on the enhancement of wound healing using antimicrobial loaded electrospun scaffolds/dressings [12,13,14,15], those were in large part based on synthetic polymers, as polycaprolactone [12,13,14] or polyethylene glycol [15], and produced using critical solvents such as formic acid [12], or chloroform [15].

Furthermore, biomaterial-based complex nanostructures developed by electrospinning could lead to great advancements in the drug delivery and bioengineering/biomedical panorama [16]. In fact, electrospinning is a robust and on-demand process with high-throughput capable of making available broadly used drugs, such as antibiotics/chemotherapeutics, and enhancing their activities thanks to the nanostructure. Moreover, the electrospun materials are characterized by high mimicry and mechanical properties capable of modulating biological processes and determining cell fate, as the case of biochemical signals [17].

## 2. Materials and Methods 

### 2.1. Materials

Chitosan (CH) (β-(1-4)-linked *D*-glucosamine and *N*-acetyl-d-glucosamine) with a low molecular weight of 251 kDa, deacetylation degree 98%, (ChitoClear, Giusto Faravelli, Milan, Italy); chondroitin sodium sulfate (CS) (β-1,4-linked d-glucuronic acid and β-1,3-linked N-acetyl galactosamine) bovine 100 EP, with a low molecular weight of 14 kDa, mixture of chondroitin A (chondroitin 4 sulfate) and chondroitin C (chondroitin 6 sulfate) (Bioiberica, Barcellona, Spain); hyaluronic acid (HA) (based on β -1,3-linked N-acetylglucosamine and β-(1,4)-d-glucuronic acid) with a low molecular weight of 212 kDa (Bioiberica, Barcellona, Spain); and pullulan (PUL) (based on maltotriose repeating units, linear α 1-4 and α 1-6 glucan, produced by *Aureobasidium pullulans*) with a low molecular weight of ~200–300 kDa (food grade, Hayashibara, Japan, Giusto Faravelli, Milan, Italy) were used for the scaffold preparations. Citric acid (CA) (monohydrated citric acid, European Pharmacopeia grade, Carlo Erba, Milan, Italy) was used as a crosslinking agent. Norfloxacin (N) (Sigma-Aldrich, Milan, Italy) was used as an antimicrobial drug.

### 2.2. Methods

#### 2.2.1. Preparation of the Polymer Blends

All the polymeric blends were based on: PUL, CH and CA; PUL, CH and CA containing CS or HA. PUL solution was prepared in distilled water and CS or HA were added to PUL, thus preparing three different solutions: PUL; PUL/CS and PUL/HA. N, as a free drug, or loaded in a hybrid system (H) (nanocomposite based on VHS and N [8]), was mixed with PUL, PUL/CS or PUL/HA. Then, CH was hydrated in acetic acid and CA was added. Three different polymeric blends were prepared by mixing each PUL, PUL/CS, and PUL/HA with CH solution at 1:1 weight ratio and norfloxacin concentration was 0.15% or 0.30% *w/w*, respectively, corresponding to 1% or 2% *w/w* in dry systems, after electrospinning. The composition of the blends prepared is reported in Table 1.

#### 2.2.2. Electrospinning Process

The polymer blends were electrospun using an electrospinning apparatus (STKIT-40, Linari Engineering, Pisa, Italy), equipped with a high voltage generator (5–40 kV), a glass syringe of 10 mL with a stainless steel needle (0.8 mm), a volumetric pump (Razel R99-E) and a planar collector. The following parameters were used to obtain N loaded scaffolds: DV (voltage) = 22 kV, needle-to-collector distance = 24 cm, flow = 0.379 mL/h, to obtain H loaded scaffolds: DV (voltage) = 24 kV, needle-to-collector distance = 22 cm, flow = 0.379 mL/h, relative humidity: 40%, environmental temperature: 25 °C. All the scaffolds were then crosslinked by heating at 150 °C for 1 h in a tight container protected from light; the process was also reported as being able to dry sterilize the products [18]. Preliminarily, N stability in the heating process was assessed using a diode array detector (DAD) HPLC (see Section 2.2.5.1). For this purpose, the active ingredient was subjected to the heating treatment in the same conditions as the scaffolds (150 °C for 1 h) (in a tight container protected from light). Chromatograms and UV/visible spectra (200–700 nm) at the maximum of the corresponding chromatographic peaks were compared.

#### 2.2.3. Chemico-Physical Characterization

Scaffold morphology was analyzed using scanning electron microscopy (SEM, Tescan, Mira3XMU, Brno, Czechia, CISRIC, University of Pavia, Pavia, Italy) after graphite sputtering in a vacuum. Nanofiber diameters were measured (Image J, ICY, Institute Pasteur, Paris, France). The presence of nanocomposite (VHS-Ns) loaded into the hybrid scaffolds was investigated by ultra-high-resolution transmission electron microscope (HR-TEM, FEI Titan G2 60–300, Thermo Fisher, Barcellona, Spain), coupled with analytical electron microscopy (AEM), with a SUPER-X silicon-drift windowless energy-dispersive X-ray spectroscopy detector. X-ray chemical element maps were collected. The samples were directly deposited onto copper grids (300 mesh coated by formvar/carbon film, Agar Scientific, Rome, Italy).

X-ray powder diffraction (XRPD) analysis was carried out using a diffractometer (X-Pert Pro model, Malven Panalytical, Monza, Italy) equipped with a solid-state detector (X-Celerator) and a spinning sample holder. The diffractogram patterns were recorded using random oriented mounts with CuKα radiation, operating at 45 kV and 40 mA, in the range 4°–60° 2*θ*. The diffraction data were analyzed using the XPOWDER^®^ software (Version 2017).

Fourier transform infrared spectroscopy (FTIR) spectra of the samples were recorded using spectrophotometer (Spectrum BX FTIR, PerkinElmer, Milan, Italy). All analyses were performed from 400 to 4000 cm^−1^ with a resolution of 0.25 cm^−1^. The results were processed with a software package (Spectrum, Perkin Elmer, I). In the Supplementary Materials, the spectra of the pristine components are reported (Figure S1).

#### 2.2.4. Mechanical Properties

Mechanical properties were assessed using a texture analyzer (TA-XT plus, Stable Microsystems, Enco, Spinea, Italy) equipped with a 1 kg load cell and A/TG tensile grips [19]. Rectangular portions (3 × 1 cm) of each scaffold (thickness ~100 μm) were kept vertical by means of two grips, the lower one fixed and the upper one movable at a constant rate of 0.5 mm/s. Dry or hydrated scaffolds were stretched up to break and the force was recorded as a function of the movable grip displacement.

The force at break was recorded and elongation % was calculated as follows:

*E*% = 100 × (*L*_break_ − *L*_0_)/*L*_break_, (1)

where L_break_ = the distance of the two grips at scaffold breaking and L_0_ = the initial distance of the two grips.

Moreover, the Young’s modulus (mN/cm^2^) was calculated as the slope of the initial linear portion of force vs. grip displacement.

#### 2.2.5. Norfloxacin Release Measurements

All the release measurements were performed in sink conditions to study drug liberation from the systems independent of the concentration of the drug released during the test [20]. Two different approaches were considered: in the first one, the drug release was studied using saline solution to simulate the lesion exudates, while in the second one, the effect of lysozyme on drug release was analyzed. Each scaffold was placed in 3 mL of dissolution medium to simulate the small number of exudates generally present in the wound, and the scaffold was completely dipped in the dissolution medium to simulate the implant of the system in the lesion bed. For this purpose, two different media were considered: saline solution (NaCl 0.9% w/v) or phosphate buffer 0.05 M (pH 6.2) containing 3.3 mg/mL of lysozyme (120.530 IU/mg, Sigma-Aldrich, Milan, Italy). As for saline solution, at prefixed times, 500 μL of dissolution medium was collected and replaced with fresh medium to keep the volume constant. The samples were analyzed by means of the DAD–HPLC method (Section 2.2.5.1) [21]. When lysozyme was present, the dissolution medium was totally collected and completely substituted with fresh medium every 24 h to avoid a loss of the enzyme activity over time. Each sample was divided in two aliquots. One aliquot was assayed to quantify the norfloxacin released from each scaffold (Section 2.2.5.1) and, for this purpose, each sample was pre-processed by diluting 1:1 with 1 N perchloric acid and by centrifugation (5000 rpm for 15 min), to precipitate the lysozyme in the solution. The second aliquot was assayed to quantify the glucosamine release, as product of lysozyme activity (Section 2.2.5.2). Moreover, the morphology of scaffolds subjected to lysozyme degradation (after 10 days) was analyzed using SEM as previously described.

##### 2.2.5.1. Norfloxacin Assay

Norfloxacin released from each scaffold was determined by DAD–HPLC (Series 200 system, PerkinElmer, Milan, Italy). A Zorbax Eclipse XDB-C8 column (4.6 mm × 150 mm, silica particle size 5 μm, Agilent, Milan, Italy) was used as the stationary phase. The mobile phase was based on acetonitrile/methanol/citric acid 0.4 M, 7:15:78 (% *v/v*) at a flow rate of 1.0 mL/min, using 275 nm wavelength detection [21,22]. The injection volume was 10 μL. Calibration curves were obtained using norfloxacin standard solution in the mobile phase, in saline solution or processed as the samples subjected to lysozyme degradation. In every case, the method was linear from 0.08 to 200 μg/mL with an R^2^ value that was always higher than 0.995.

##### 2.2.5.2. Glucosamine Assay

Glucosamine released due to the lysozyme degradation of the scaffolds was quantified by means of ninhydrin assay [23].

All samples were diluted 1:1 ratio (*v/v*) with 400 µL of ninhydrin reagent (ninhydrin 2% *w/v*, hydrindantin 6.8 mg/L in 3:1 *v/v* dimethylsulfoxide: lithium acetate buffer 4 M, pH 5.2; Sigma-Aldrich, Milan, Italy) under a nitrogen blanket. Each sample was stirred at 100 °C for 8 min, and vortexed until cooling, then the samples were diluted 1:10 (*v/v*) with a 1:1 ethanol:water mixture and quantified by a colorimetric test at ʎ = 570 nm using an ELISA Plate Reader (iMARK Microplate Absorbance Reader, BioRad, Milan, Italy). The calibration curve (glucosamine in phosphate buffer 0.05 M at pH 6.2) was linear in the range from 0.0125 to 0.1 μg/mL with a *R*^2^ > 0.995.

#### 2.2.6. Biopharmaceutical Characterizations

Adhesion and proliferation assay was carried out using normal human dermal fibroblasts (NHDF) from juvenile foreskin (PromoCell, VWR, Milan, Italy) [9,10]. Fibroblasts were grown in the presence of 150 µL Dulbecco’s modified Eagle medium (DMEM, Sigma-Aldrich, Milan, Italy) supplemented with 10% *v/v* fetal bovine serum (Euroclone, Milan, Italy), and with penicillin/streptomycin solution (pen/strep, 100 UI/100 μg/mL, Sigma-Aldrich, Merck, Milan, Italy), at 37 °C in a 5% CO_2_ atmosphere with 95% relative humidity. The 0.36 cm^2^ circular portion scaffolds were placed at the bottom of the wells in a 96-well plate (flat bottom, Cellstar©, Greiner bio-one, Frickenhausen, Germany). Fibroblasts were seeded onto the scaffolds at a seeding density of 35,000 cells/well and grown for 3 or 6 days. The cell growth without scaffolds (35,000 cells/well) was considered the standard growth (growth medium (GM)). After 3 or 6 days, the MTT [3-(4,5-dimethylthiazol-2-yl)-2,5-diphenyltetrazolium bromide] assays were performed. The fibroblasts that adhered and grew onto the scaffolds (growth for 6 days) were fixed for 2 h at 4 °C, using 3% *w/v* of glutaraldehyde in Dulbecco’s phosphate buffered saline (PBS, Sigma-Aldrich, Milan, Italy) and analyzed by SEM and confocal laser scanning microscopy (CLSM), as described in the following paragraphs.

##### MTT Assay

The biocompatibility was performed by MTT test (tetrazolium salt, [3-(4,5-dimethylthiazol-2-yl)-2,5-diphenyltetrazolium bromide]; Sigma-Aldrich, Milan, Italy). Briefly, MTT was solubilized at 2.5 mg/mL in PBS (phosphate buffer solution, Sigma-Aldrich, Milan, Italy). At prefixed days, the medium in each well was removed and 50 μL of MTT solution plus 100 μL of PBS were added and subsequently put in contact with the cell substrates at 37 °C for 3 h in the incubator. Then, MTT solution was removed from each well and 100 μL of dimethylsulfoxide (DMSO, Sigma-Aldrich, Milan, Italy) was added. The absorbance was read using an ELISA Plate Reader at ʎ = 570 nm (with reference ʎ = 690 nm).

##### SEM Analysis

The substrates were then washed three times with PBS and dehydrated with ethanol solutions at increasing concentrations (50–75–100% *v/v*). The scaffolds were then removed from culture wells, applied onto stubs, sputtered with graphite and analyzed by SEM, as previously described.

##### CLSM Analysis

The substrates were then washed three times with PBS. Then cell actin cytoskeleton was stained with phalloidin FITC Atto 488 (50 μL at 20 μg/mL in PBS in each well, contact time 30 min) (Sigma-Aldrich, Milan, Italy). Subsequently, after three PBS washes, the cell nuclei were stained with Hoechst 33258 (100 μL of solution at 1:10,000 dilution in PBS per each well, contact time 10 min in the dark) (Sigma-Aldrich, Milan, Italy), for 10 min. After three further PBS washes, the scaffolds were mounted on glass slides, covered using coverslips and analyzed using CLSM (Leica TCS SP2, Leica Microsystems, Milan, Italy) at λ_ex_ = 346 nm and λ_em_ = 460 nm for Hoechst 33258 and λ_ex_ = 501 nm and λ_em_ = 523 nm for phalloidin FITC. The acquired images were processed by means of Leica software (Leica Microsystem, Milan, Italy).

#### 2.2.7. In Vitro Antimicrobial Assay

The antimicrobial activity of norfloxacin-loaded scaffolds, either as free drug, N, or in nanocomposite, H, was evaluated against two bacteria strains—*Staphylococcus aureus* ATCC 6538 and *Pseudomonas aeruginosa* ATCC 15442. In particular, killing time was determined as the exposure time required to kill a standardized microbial inoculum [8,10,23]. Bacteria used for killing time evaluation were grown overnight in Tryptone Soya Broth (Oxoid, Basingstoke, Hampshire, UK) at 37 °C. The bacteria cultures were centrifuged at 2000 rpm for 20 min to separate the cells from the broth and then suspended in phosphate buffer saline (PBS, pH 7.3). The suspension was diluted to adjust the number of cells to 10^7^–10^8^ CFU/mL (CFU = colony forming unit).

For each microorganism strain, a suspension was prepared in PBS without scaffolds and used as the control. Unloaded scaffolds were also tested for comparison. Bacterial suspensions were incubated at 37 °C. Viable microbial counts were evaluated after contact for 0, 5, and 24 h with scaffolds and in control suspensions; bacterial colonies were enumerated in Tryptone Soya Agar (Oxoid, Basingstoke, Hampshire, UK) after incubation at 37 °C for 24 h. The microbiocidal effect (ME value) was calculated for each test organism and contact times were calculated according to the following equation [10,23]:

ME = log Nc − log Nd,(2)

where Nc is the number of CFUs in the control microbial suspension and Nd is the number of CFUs in the microbial suspension in the presence of the scaffold.

#### 2.2.8. Statistical Analysis

Statistical differences were evaluated by means of a one-way ANOVA post-hoc Fisher’s Least Significant Difference (LSD) or Mann–Whitney (Wilcoxon) W test (Statgraphics Centurion XV, Statistical Graphics Corporation, Statgraphics Technologies, Inc., The Plains, Virginia, USA). Differences were considered significant at *p* < 0.05.

## 3. Results and Discussion

### 3.1. Chemico-Physical Characterization

**Figure 1:**
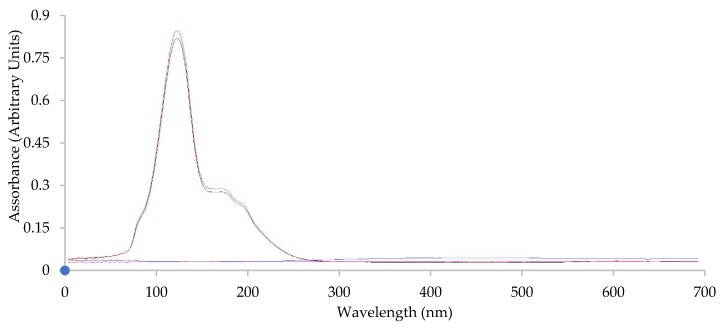
UV spectra of N (red line) and N subjected to heat treatment (1 h at 150 °C) (black line) obtained from the HPLC analysis at N peak maximum.

Preliminarily, to scaffold preparation and cross-linking by heating, the drug stability in the heating process was assessed. For this purpose, the drug was subjected to a heating treatment in the same conditions used for the scaffold cross-linking (1 h at 150 °C). The heating treatment did not cause the drug degradation; in fact, after the process, the N content was 99.21% *w/w* (SD = 2.04) compared to the active ingredient in standard storage conditions. Figure 1 reports the UV spectra of N, and N subjected to the heating treatment at the maximum of the chromatographic peak (N retention time).

The complete overlapping of the spectra supported the stability of the drug in the heating treatment.

In a previous work [8], norfloxacin was loaded in montmorillonite, a phyllosilicate widely used in pharmaceutical field, to obtain a nanocomposite. This was prepared by means of the adsorption mechanism, as one single process, and the clay–drug adsorption isotherm was calculated. The solid-state analysis (XRPD, FTIR, thermal analysis—differential scanning calorimetry/ thermogravimetric analysis DSC/TGA, HRTEM) evidenced that protonated norfloxacin molecules interact with the active sites of montmorillonite located at its edges and within its interlayer space, thus forming a drug monolayer onto the clay mineral interlayer surface. Norfloxacin in the nanocomposite was proved in an amorphous state, and its loading (16% *w*/*w* of total nanocomposite weight) is homogeneous and causes an expansion of montmorillonite interlayer spaces. Moreover, the nanocomposite causes a prolonged norfloxacin release over time. Moreover, the nanocomposite was characterized by good biocompatibility in vitro toward fibroblasts, and it was able to increase the antimicrobial potency of the free drug against *P. aeruginosa* and *S. aureus*, Gram-negative and Gram-positive bacteria, respectively, both of which are often concurrent causes of wound chronicization, leading to the possible impairment of the healing path and, finally, to nonhealing wounds. Montmorillonite norfloxacin nanocomposite was loaded into scaffolds and their performance was compared to those loaded with the free drug scaffolds.

**Figure 2:**
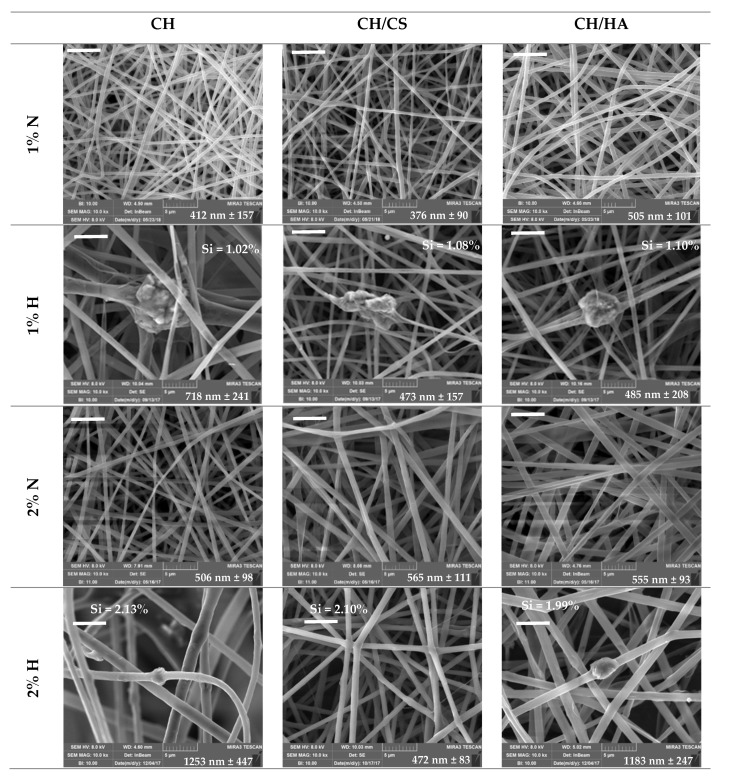
SEM microphotographs of chitosan-based (CH), chitosan/chondroitin sulfate-based (CH/CS) and chitosan/hyaluronic acid-based (CH/HA) scaffolds loaded with 1% or 2% norfloxacin, as a free drug (1% N or 2% N), or in VHS-N nanocomposite. In each image, the nanofiber diameters (nm, mean values ± SD; n = 30) and Si content for hybrid scaffolds are reported. Statistics: Mann–Whitney (Wilcoxon) W test *p* < 0.05: CH2H vs. CH/CS2H; CH/CS2H vs. CH/HA2H; CH/HA1H vs. CH/HA2H; CH/HA2H vs. CH/HA2N (scale bar 5 μm).

Figure 2 reports SEM microphotographs of CH, CH/CS or CH/HA scaffolds loaded with 1% or 2% norfloxacin, as a free drug (1% N or 2% N), or loaded with VHS-N nanocomposite (1% H or 2% H).

The N scaffolds, loaded with N as a free drug, were characterized by a regular structure with a smooth surface where no ribbon could be detected, independent of drug concentration. The H scaffolds, loaded with N in nanocomposite, presented nanofiber portions with a regular, smooth surface spaced out in broadened parts, with knots and a scattered structure. These conceivably could be related to the montmorillonite–norfloxacin (VHS-N) nanocomposite. Moreover, the presence of glycosaminoglycans (CS or HA) in the scaffolds caused a certain degree of surface roughness (probably due to chitosan and glycosaminoglycan interaction [9]) and this was more evident due to the increasing drug concentration in the fibers.

Nanofiber diameters were generally smaller when norfloxacin was loaded as a free drug (around 500 nm), independent of the drug concentration, although the differences were not statistically significant. On the contrary, H scaffolds were characterized by nanofibers with higher diameters (around 500 nm for 1% scaffold and around 1000 nm for 2% scaffolds) compared to those containing 1% of the drug, although this was significant only for the CH/HA scaffold; in this case, HA’s high molecular weight was ten folds greater than that of CS and could cause the formation of fibers with greater diameters. On the contrary, H scaffold containing chondroitin sulfate and loaded with 2% of the drug showed similar nanofiber diameters to those loaded with the free drug. The content of Si, an element characteristic of montmorillonite, was consistent with the nanocomposite concentration in each scaffold [23].

The analysis of system viscosity previously performed on the blank systems [9], stated that chondroitin sulfate (negatively charged) conceivably interacted with chitosan (positively charged) and this could be due to the high charge density of sulfate groups greater than those of the carboxylic moieties of hyaluronic acid. However, the presence of particles in suspension, as was the case in nanocomposite, could cause unbalanced particle charge density that generally increases the conductivity, influencing fiber diameter during electrospinning [24]. Moreover, the acid environment of the polymer blends, due to the 45% *v/v* acetic acid in the medium, conceivably prevented the interactions between the various moieties and drug precipitation [25,26].

**Figure 3:**
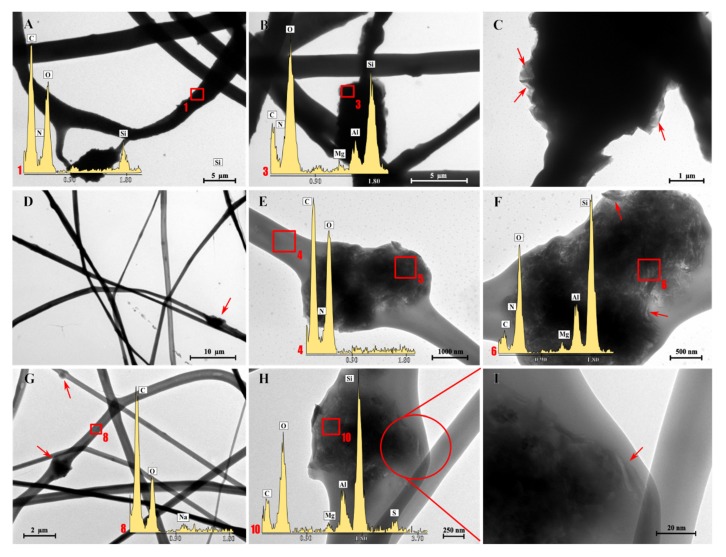
Transmission electron microscope (TEM) microphotographs and EDX spectra for CH, CH/CS and CH/HA scaffolds loaded with 2% norfloxacin in nanocomposite CH (**A**–**C**), CH/CS (**G**–**I**) and CH/HA (**D**–**F**).

Figure 3 reports the HR-TEM microphotographs and EDX spectra obtained for CH (A–C), CH/CS (G–I) and CH/HA (D–F) H scaffolds, loaded with N in the nanocomposite at 2%.

The EDX analysis performed in the marked zone (red square) confirms that there was the presence of C, O and N (typical of organic elements) and characteristic elements of montmorillonite (Si, Al, Mg) in the broad, interwoven knots. This was observed for all the scaffolds, independent of their polymeric composition. At a higher magnification (Figure 3C,F,I), it was possible to identify the typical lamellar structure of montmorillonite (red arrows).

**Figure 4:**
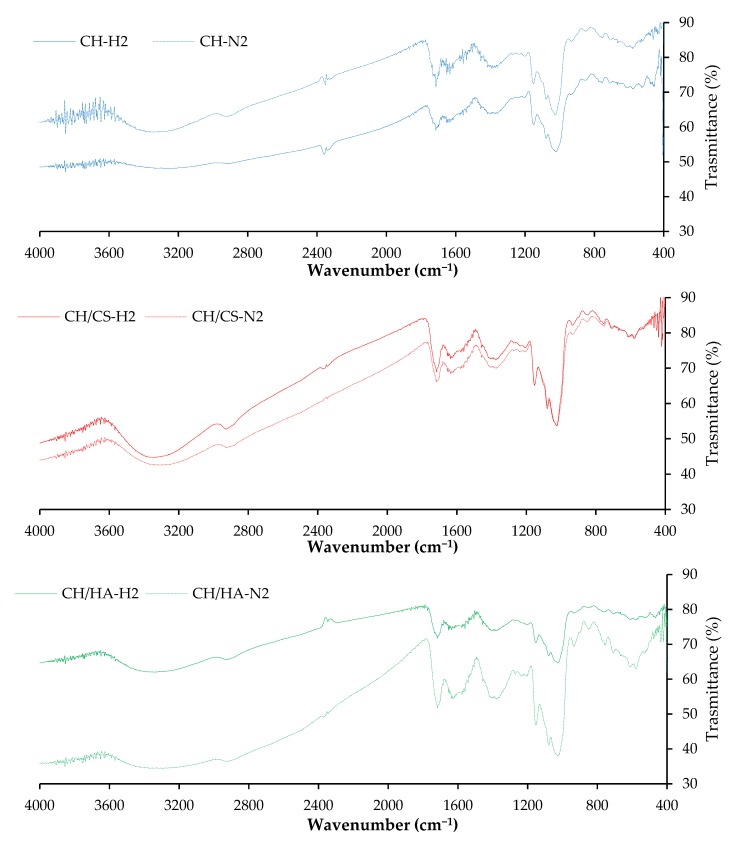
Fourier transform infrared spectroscopy (FTIR) spectra evaluated for norfloxacin-loaded scaffolds (CH-N2, CH/CS-N2, CH/HA-N2) and VHS-N loaded scaffolds (CH-H2, CH/CS-H2, CH/HA-H2), both types containing 2% *w/w* norfloxacin in norfloxacin–montmorillonite nanocomposite (VHS-N).

Figure 4 reports FTIR spectra evaluated for norfloxacin-loaded scaffolds (CH-N2, CH/CS-N2, CH/HA-N2) and VHS-N loaded scaffolds (CH-H2, CH/CS-H2, CH/HA-H2), both types containing 2% *w/w* norfloxacin.

Independent of the polysaccharide composition and loading type, either with N (free drug, norfloxacin) or H (VHS-N nanocomposite), the typical polysaccharide signals (hydrogen bonds of –OH and –NH_2_ groups) (pullulan: 3331 cm^−1^ and chitosan: 3355 cm^−1^) hid the drug and nanocomposite-related peaks [27]. In fact, the VHS spectrum should present a band around 1017 cm^−1^ due to the vibrational band of the silicates.

**Figure 5:**
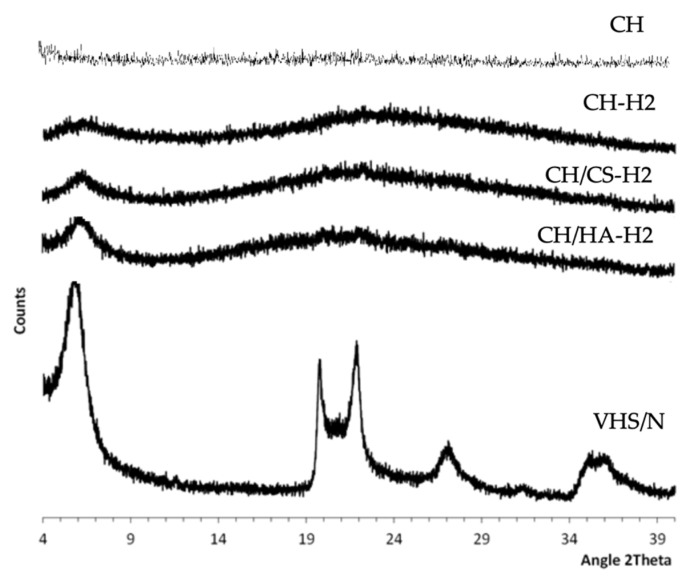
X-ray powder diffraction (XRPD) diffractograms of the scaffolds loaded with VHS-N at 2% norfloxacin (CH-H2, CH/CS-H2, CH/HA-H2) compared to unloaded CH scaffolds (CH) and VHS-N.

Figure 5 reports the XRPD patters of the scaffolds loaded with VHS-N nanocomposite containing norfloxacin at 2% compared to VHS-N, the nanocomposite and the unloaded CH scaffold.

All the unloaded scaffolds were characterized by amorphous behavior (CH pattern is reported in Figure 5 as example) and no paracrystallinity could be detected. This was probably related to the electrospinning process. For all the scaffolds, the diffractograms were characterized by a hump between 20° and 29° 2*θ*, which was probably due to the presence of the polysaccharides. The reflection peak centered at 6° 2*θ*, which was probably due to the nanocomposite (VHS-NF) since it coincided with the d001 of montmorillonite once NF located in the interlayer space (5.94° 2*θ*). Similar XRD results were obtained by Rabbani et al. (2016) [28]. Moreover, there was an absence of other intense peaks that could be attributable to the nanocomposite (VHS-N), probably due to the nanocomposite concentration in the scaffolds, which was too low.

### 3.2. Mechanical Properties

**Figure 6:**
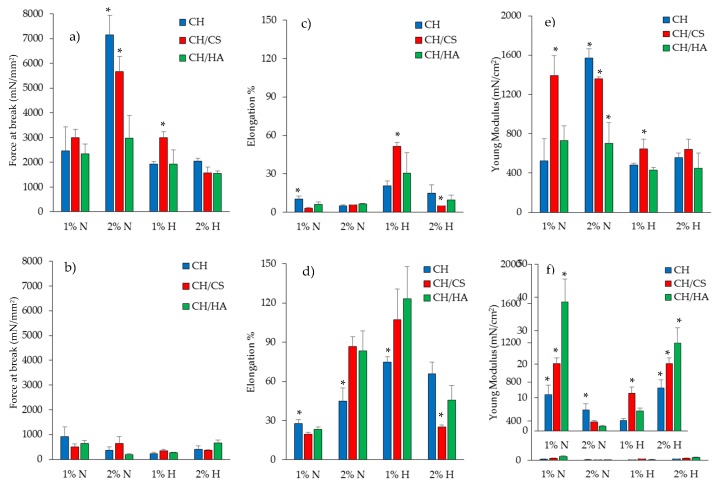
Mechanical properties (force at break mN, a-b; elongation %, c-d; Young’s modulus mN.cm^2^, e-f) for dry (**a**,**c**,**e**) and wet (**b**,**d**,**f**) scaffolds loaded with 1% (**a**) or 2% (**b**) of norfloxacin as a free drug (N) or as nanocomposite (H) (mean values ± SD; *n* = 3). Statistics: * = Mann–Whitney (Wilcoxon) W test *p* < 0.05.

Figure 6 reports the mechanical properties (force at break mN, a–b; elongation %, c–d; Young’s modulus mN·cm^2^, e–f) of scaffolds loaded with 1% or 2% of norfloxacin as a free drug (N) or as a nanocomposite (H), in dry (a, c, e) or wet (b, d, f) conditions.

In a dry state, the increase in N concentration in the scaffolds caused an increase in the force at break, except for the scaffold containing hyaluronic acid (Figure 3a). This was less evident when norfloxacin was loaded as a nanocomposite: it is conceivable that the effect of montmorillonite, which altered the entanglement of polymer chains in the scaffolds, causing lower resistance to break, prevailed over the effect attributable to the free drug, which seems to reinforce the structure. In this condition, the N scaffolds were less deformable than H scaffolds and the N concentration at 1% in H scaffolds was responsible for a higher deformability (Figure 3c). Moreover, the free drug seems to increase scaffold elasticity, especially for scaffolds containing chondroitin sulfate (Figure 3e). The hydration of the scaffolds, which simulates the application/implant in the lesion, dramatically changed the scaffold mechanical properties. N scaffolds, loaded with N as a free drug, were characterized by slightly higher resistance to break with respect to H scaffolds, confirming the behavior of the dry state (Figure 3b), while the scaffolds were simultaneously characterized by a higher degree of deformability (Figure 3d), which could be advantageous for wound bed application, and low elasticity (Figure 3f). The hydration caused a remarkable decrease in resistance to break, an increase in deformability and a loss of elasticity.

The presence of montmorillonite in the hybrid scaffolds seems to weaken the scaffold structure, and this was probably due to the presence of particles embedded into the polymeric matrix that could disrupt the polymer chain entanglements, causing a significant decrease in the scaffold elasticity, and mechanical resistance, and a directly related increase in the deformability: this was more evident when 2% of drug in the nanocomposite was loaded in the scaffolds compared to scaffolds loaded with the free drug.

The mechanical properties are key features for the success of scaffold implants and their integration with the surrounding tissue. In fact, the native skin is characterized by tensile strength values approximately between 5.0 and 30.0 MPa (5000–30,000 mN/mm^2^), the Young’s modulus in the range of 4.6–20.0 MPa (46–200 mN/cm^2^) and the elongation at break of about 35.0–115.0% [29]. Clearly, the ranges of the reference values are wide since the mechanical properties of the skin are strictly related to age and body lines (static lines, as described by Langer, Kraissl’s lines or Borge’s lines) [30]. In particular, force at break (mechanical strength) is related to the scaffold’s capability to maintain its integrity during implantation, which should occur in the dry state, while the elongation and the Young’s modulus are mainly related to the scaffold performance upon implantation. The scaffolds developed in the present work were characterized by force at break in the dry state close to the skin, especially for CH and CH/CS scaffolds, when loaded with norfloxacin at 2% as a free drug. Moreover, upon hydration, all the scaffolds were characterized by elongation superimposable to that of native skin. Furthermore, as for the Young’s modulus, the scaffolds were characterized by the stiffness/elasticity closest to that of the skin, both in dry and hydrated states. Moreover, there is evidence in the literature that correlates the fibroblast adhesion and proliferation to substrate stiffness [31]; stiff matrices with a 2 MPa Young’s modulus enhanced fibroblast proliferation much more than an elastic substrate (0,042 MPa). In fact, in the literature, there is evidence that the fibroblasts of granulation tissue are proliferative and motile, while those of the dermis are in a quiescent and stationary state [32]. Moreover, stiff substrates were demonstrated to sustain cell spreading and to facilitate guiding the pro-angiogenic signaling of fibroblasts [33].

### 3.3. Norfloxacin Release Properties

**Figure 7:**
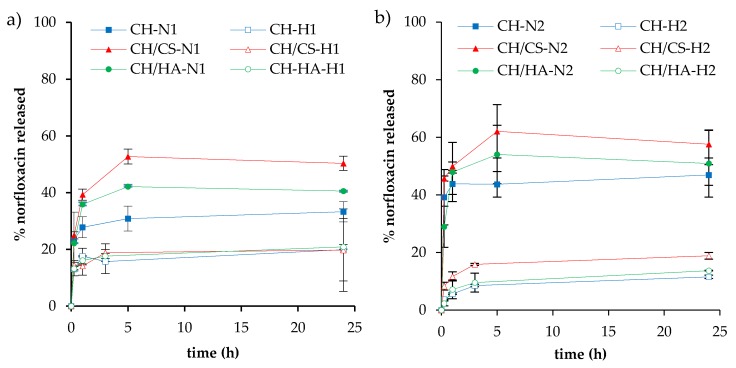
Release profiles (%) of norfloxacin from the N or H scaffolds loaded with 1% (**a**) or 2% (**b**) as a free drug (N) or as nanocomposite (H), in saline solution (mean values ± SD; *n* = 3).

Figure 7 reports the release profiles of norfloxacin in saline solution. As for H scaffolds (N loaded as nanocomposite), independent of the drug loading, the profiles reached plateau values at 20% of the drug released after 3 h.

As for N scaffolds (N loaded as a free drug), independent of the drug loading, the release profiles reached plateau values after 5 h; CH/CS scaffolds were characterized by their higher profile (50% and 57% for 1% and 2% N loading, respectively) followed by CH/HA scaffolds (about 40% and 50% for 1% and 2% N loading, respectively) and finally by CH scaffolds (33% and 50% for 1% and 2% N loading, respectively). When norfloxacin was loaded in the scaffolds as a nanocomposite (H scaffolds), the release was lower than when the scaffolds contained the free drug, and this seems to be independent of scaffold polymer composition. On the contrary, when norfloxacin was loaded as a free drug (N scaffolds), the presence of glycosaminoglycans markedly influenced norfloxacin release. This could be due to an interaction between anionic glycosaminoglycans and cationic chitosan forming a polyelectrolyte complex, which could make the fibrous structure less entangled and, therefore, more available to interact with the dissolution medium and to allow drug diffusion through the polymer matrix and, consequently, its release. In fact, scaffolds containing chondroitin sulfate, characterized by a charge density greater than hyaluronic acid, were characterized by a higher release profile. Chondroitin sulfate is characterized by the sulfate groups having an acid behavior greater than the carboxylic groups of hyaluronic acid. Consequently, the interaction between chondroitin sulfate and chitosan could cause a coiled structure less prone to polymer chain entanglements [34]. 

In any case, in the scaffolds loaded with higher concentrations of the drug, this difference was less evident with respect to those with lower drug loadings.

**Figure 8:**
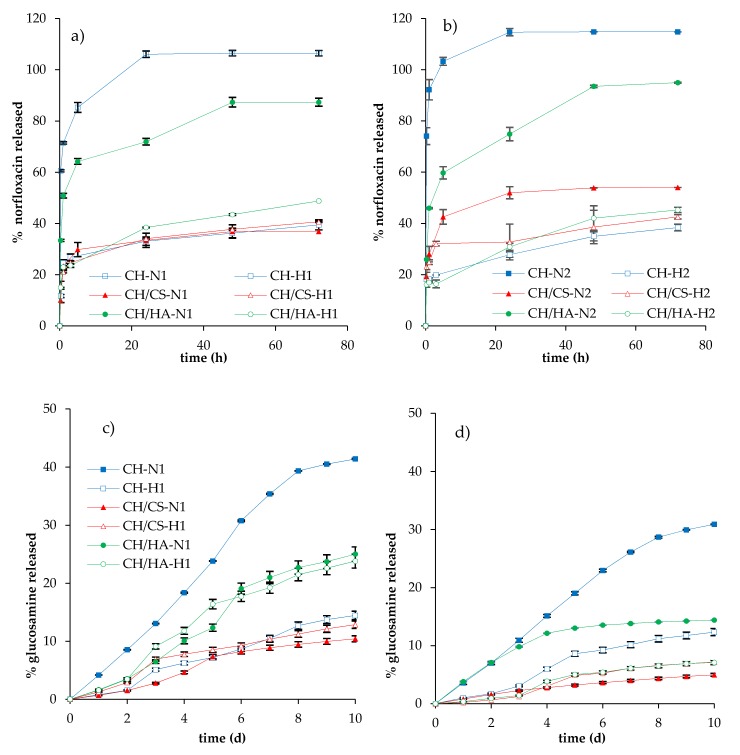
Norfloxacin released (%) in lysozyme from the scaffolds loaded with 1% (**a**) or 2% (**b**) of norfloxacin as a free drug (N) or as nanocomposite (H) and glucosamine released (%) from the scaffolds loaded with 1% (**c**) or 2% (**d**) of norfloxacin as a free drug (N) or as nanocomposite (H) subjected to lysozyme activity (mean values ± SD; n = 3)

Figure 8 reports the norfloxacin release profiles (a and b) and glucosamine release profiles (c and d) of scaffolds subjected to lysozyme degradation. 

**Figure 9:**
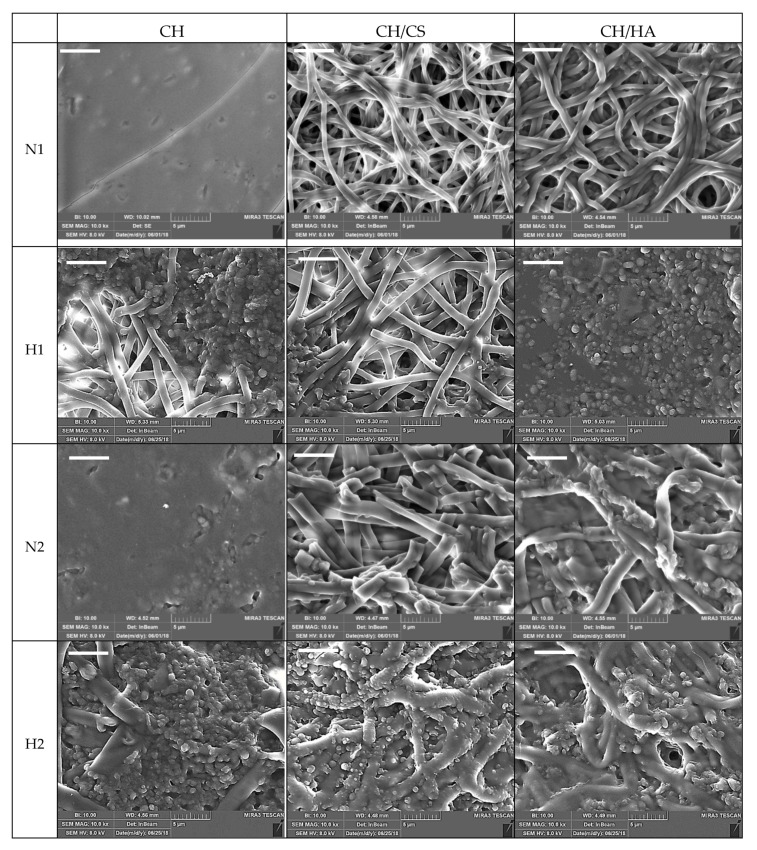
SEM microphotographs of the scaffolds loaded with 1% or 2% of norfloxacin as a free drug (N) or as nanocomposite (H) subjected to lysozyme activity for 10 days (scale bar: 5 µm).

Independent of norfloxacin concentration in the N scaffolds (free drug loading), the activity of lysozyme markedly increased the drug release: CH scaffolds containing chitosan, without glycosaminoglycans, showed higher release profiles, reaching, in almost 24 h, 100% of the drug being released; scaffolds based on CH/HA showed 80% of the drug being released in 48 h, while CH/CS scaffolds were characterized by a lower release close to 40–50% of the drug being released in 72 h, for 1% or 2% norfloxacin loading, and these profiles were similar to those obtained in saline solution. These behaviors could be explained considering that the activity of lysozyme on the scaffold matrices: CH scaffolds completely lost their nanofibrous structure in contact with lysozyme (Figure 9). On the contrary, after 10 days of lysozyme activity, the CH/CS scaffold and, mainly, the CH/HA scaffold showed a residual of nanofibrous structure, submerged in a non-structured material. It is conceivable that the interaction of chitosan amino groups (positively charged) with either sulfate groups of chondroitin sulfate or the carboxylic ones of hyaluronic acid (both negatively charged) conferred a higher resistance against enzyme degradation, probably hindering interaction with the substrate. Moreover, chitosan/glycosaminoglycan interactions could partially prevent the loss of the system morphology, decreasing drug release.

In hybrid H scaffolds, loaded with norfloxacin in the montmorillonite nanocomposite, the profiles of norfloxacin released in the presence of the lysozyme were higher than those obtained in saline solution, although no difference could be evidenced, considering both the scaffold composition and the percentage of drug loaded, and all the scaffolds were characterized by release profiles reaching drug loading of 50% in 72 h.

However, in all cases, the glucosamine release profiles suggest that the enzymatic degradation of chitosan occurred, independent of system composition and percentage of drug loaded. CH scaffolds were characterized by their higher profiles, followed by H/HA scaffolds and CH/CS ones. Generally, the presence of norfloxacin–montmorillonite nanocomposite seems to decrease the lysozyme activity and the profiles of glucosamine (degradation product) were consistent with the norfloxacin release ones. Furthermore, the drug loading seems to have a negative impact on enzymatic activity and the glucosamine release profiles were higher in 1% loaded systems than in 2% ones. It is reported in the literature that lysozyme interacts with quinolones and this supports that there is a competition between norfloxacin and chitosan, as enzyme substrates, decreasing the enzymatic activity towards chitosan degradation when norfloxacin is at higher concentrations [35]. Moreover, the presence of montmorillonite in the scaffolds could impair lysozyme activity, probably due to a certain degree of interaction between montmorillonite and chitosan, which could prevent chitosan interaction with the enzyme. Furthermore, the interaction between chitosan and either chondroitin sulfate or hyaluronic acid could render chitosan, as lysozyme substrate, less prone to interaction with lysozyme, resulting in less efficient degradation activity towards chitosan [36].

Similar norfloxacin release profiles were observed by Dua et al. [37] for semisolid systems loaded with 1% norfloxacin. Dependent of the type of system, drug release ranged from 70% to 41% in 7 h. The highest drug release was observed for Carbopol-based gel (about 70%) followed by polyethylene glycol-based formulation (66%), HPMC-based gel (45%) and, finally, the slowest release was evidenced in the case of an ointment. Analogous behavior was observed by Denkbaş et al. [38] and Mahmoud and Salama [21] for chitosan and chitosan collagen sponge-like dressings loaded with norfloxacin. In those cases, the norfloxacin release was mainly related to system swelling that controlled the drug diffusion for an extended time of up to 4 days.

However comparing the features of the nanofibrous scaffolds presented in this work with those of the systems in the literature, the capability of the scaffolds based on chitosan or glycosaminoglycan (either chondroitin sulfate or hyaluronic acid) associated with chitosan (CH, CH/CS and CH/HA) to possess minimal swelling (as shown by SEM images after 6 days of hydration in aqueous environment) and controlled norfloxacin release, tuned up by both the hydration and the activity of lysozyme (secreted during the inflammatory phase of wound healing), confer the ideal properties of these systems. Indeed, as soon as the systems can be implanted, norfloxacin release should occur due to the hydration of exudate from the lesions; subsequently, the inflammatory phase, preceding the proliferative one, should lead to a further release of the drug to support the whole healing process.

Figure 9 reports SEM microphotographs of all the scaffolds subjected to 10 days of enzymatic degradation by lysozyme. These images are in agreement with the glucosamine release profiles (Figure 8c,d). In fact, the higher degree of scaffold degradation (loss of nanofibrous structure) was associated with a higher glucosamine release profile. Independent of drug concentrations in CH scaffolds containing chitosan, without glycosaminoglycans, and loaded with norfloxacin as a free drug, the nanofibrous structure was no longer visible, while CH scaffolds loaded with norfloxacin in nanocomposite were characterized by a nanofibrous structure, partially covered by spherical particles, reported in the literature as lysozyme molecules attached to the biopolymer matrix [36]. The presence of glycosaminoglycans in the scaffolds determined a higher resistance against enzymatic activity. In some cases, as for the CH/CS-N2 scaffold, nanofibers were partially broken, swollen, and partially fused. Long-lasting scaffold degradation could be advantageous, especially in deep/cavity wounds, since this should allow the gradual replacement of the scaffold matrix with native tissue, due to the production of the extracellular matrix by fibroblasts.

### 3.4. Cytocompatibility: Fibroblast Adhesion and Proliferation

**Figure 10:**
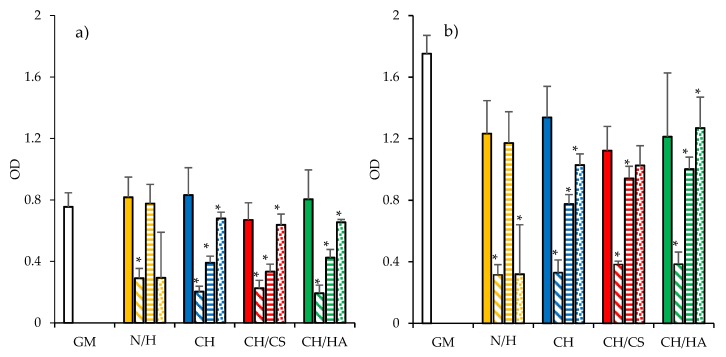
Cytocompatibility (optical density (OD)) of fibroblasts grown for 3 days (**a**) and 6 days (**b**) onto CH (blue), CH/CS (red), CH/HA (green) loaded with norfloxacin at 1% (plain color) and 2% (oblique lines), and with norfloxacin–montmorillonite nanocomposite (N-VHS) at 1% (horizontal lines) and 2% (dots) in norfloxacin. N norfloxacin as a free drug and H (N-VHS nanocomposite) at the same concentrations of the scaffolds are evaluated (mean values ± SD; n = 8). Statistics: * = Mann–Whitney (Wilcoxon) W test *p* < 0.05.

Figure 10 reports the cytocompatibility (optical density (OD)) of the scaffolds towards fibroblasts after 3 or 6 days of growth. Fibroblast adhesion and proliferation onto the scaffolds were compared to those of the control (GM and cell growth in standard conditions).

The cytocompatibility of the scaffolds loaded either with norfloxacin as a free drug or with the nanocomposite VHS-N at 1% or 2% were evaluated and N or H at the same concentrations as in the scaffolds were considered for comparison. After 6 days of growth, all the samples were characterized by higher OD than those after 3 days of growth, suggesting that the cells were in proliferation. Considering the scaffolds loaded with N as a free drug, the increase in drug concentration caused a significant decrease in cell viability with respect to the control. These results were similar to those obtained for N. This indicates that the decrease in cytocompatibility could be completely attributed to the drug and not to the scaffolds.

On the contrary, for the scaffolds loaded with the nanocomposite, the drug loading increased the cytocompatibility, suggesting that the nanocomposite in the scaffolds was able to prevent the negative effect of norfloxacin towards the fibroblasts (this was also evident considering the cytocompatibility of the nanocomposite, which was higher for the 1% solution than the 2% solution). Such an increase in cytocompatibility could be due to montmorillonite, which was able to control the drug release and could enhance fibroblast proliferation [39].

**Figure 11:**
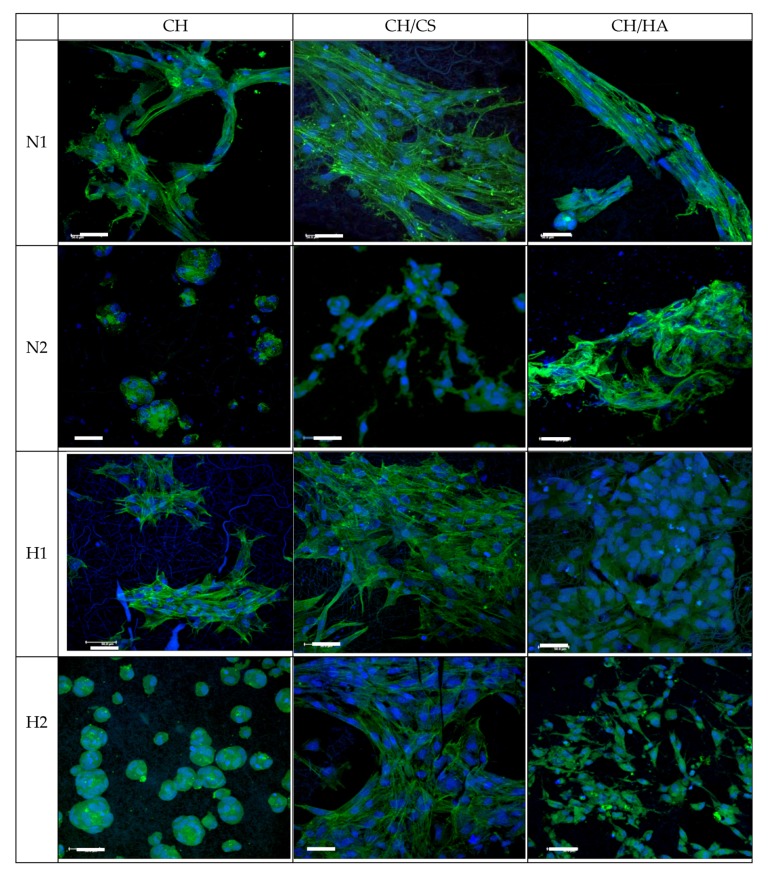
Confocal laser scanning microscopy (CLSM) microphotographs of fibroblasts grown for 6 days onto CH, CH/CS, CH/HA loaded with norfloxacin as a free drug (N) or norfloxacin–montmorillonite nanocomposite (H, N-VHS) at 1% or 2% (in blue: nuclei; in green: cytoskeleton) (scale bar: 50 μm).

**Figure 12:**
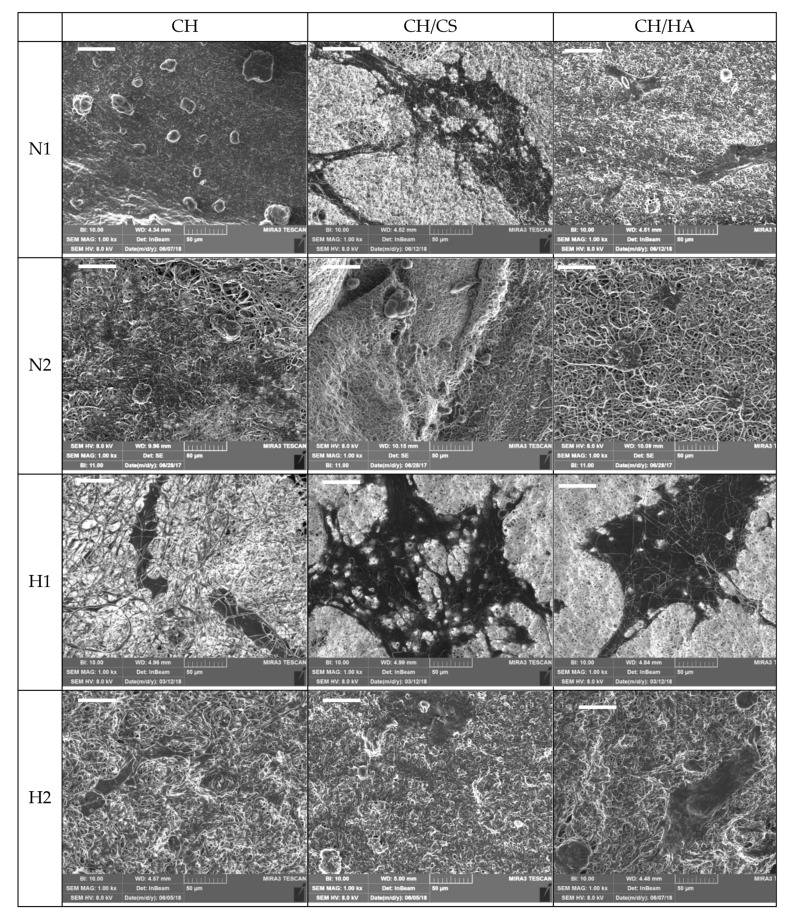
SEM microphotographs of fibroblasts grown for 6 days onto CH, CH/CS, CH/HA loaded with norfloxacin as a free drug (N) or norfloxacin–montmorillonite nanocomposite (H, N-VHS) at 1% or 2% (scale bar: 50 μm).

Figure 11 and Figure 12 report CLSM and SEM microphotographs of fibroblasts grown for 6 days onto CH, CH/CS, CH/HA loaded with norfloxacin at 1% or 2%, either as a free drug or in the nanocomposite. The complementary information from the SEM and CLSM analyses suggests that in the scaffolds loaded with N as a free drug, the fibroblasts were not homogeneously distributed on the scaffolds and mainly formed aggregates as cell clusters. This behavior was dramatically influenced by drug concentration, in agreement with the cytocompatibility. However, both the scaffolds containing glycosaminoglycans allowed the fibroblasts to maintain their fusiform structure and cytoskeletons based on aligned and elongated filaments. However, norfloxacin concentration did not alter nuclei morphology. In the hybrid scaffolds, loaded with norfloxacin nanocomposite, fibroblasts were spread out all over the scaffolds and, in some areas, confluence could be reached and, although all the scaffolds were effective to allow cell adhesion and proliferation, the scaffolds containing chondroitin sulfate were characterized by their better performance.

These results are in agreement with others in the literature stating the biocompatibility and the proliferation enhancement properties of montmorillonite and halloysite, both phyllosilicates with a planar and rolled structure, respectively [40,41,42]. Moreover, the polymer matrix of the scaffolds had a synergic effect with montmorillonite, leading to effectiveness in enhancing cell growth in the presence of norfloxacin [43].

Moreover, the mechanical properties combined with norfloxacin release could better support fibroblast adhesion, proliferation and spreading all over the scaffold when the norfloxacin is loaded in the scaffolds as a nanocomposite, at 1% concentration, and chondroitin sulfate or hyaluronic acid are present in the composition.

### 3.5. Antimicrobial Properties

**Figure 13:**
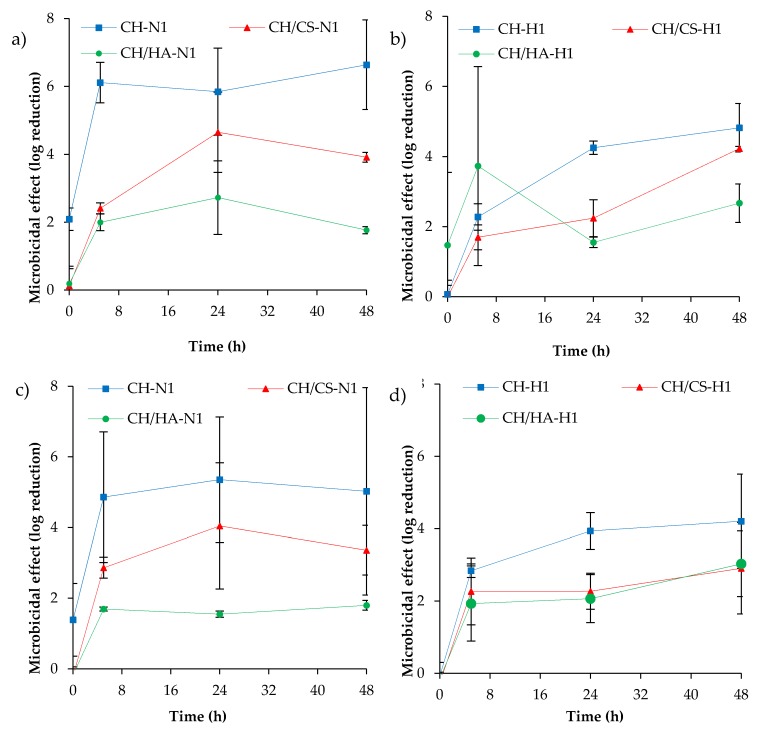
Microbicidal effect evaluated for 1% norfloxacin as a free drug (**a**,**c**) and (**b**,**d**) as nanocomposite loaded into CH, CH/CS and CH/HA scaffolds against Pseudomonas aeruginosa (**a** and **b**) and Staphylococcus aureus (**c** and **d**), in comparison to norfloxacin as a free drug and as nanocomposite, with the same concentration as in the scaffolds (mean values ± SD; n = 3).

Figure 13 reports the microbicidal effect vs. time profiles evaluated for CH, CH/CS and CH/HA scaffolds loaded with norfloxacin as a free drug (a, c) and (b, d) as nanocomposite at 1% against Pseudomonas aeruginosa and Staphylococcus aureus.

*Pseudomonas aeruginosa* is a facultative Gram-negative anaerobe bacterium. It is recognized as a multidrug-resistant pathogen for its intrinsically advanced antibiotic resistance mechanisms since it causes infections of considerable medical importance, among them hospital-acquired infections such as sepsis syndromes. *Staphylococcus aureus* is facultative Gram-positive anaerobe bacterium. It is part of the skin microbiota; however, as an opportunistic pathogen, it could cause skin infections. Moreover, *S. aureus* could become resistant to antibiotics, and its methicillin-resistant strains are a worldwide emergency in clinical medicine. N is reported in the literature as being effective against both *P. aeruginosa* and *S. aureus*, having a MIC (minimal inhibitory concentration) of 2 μg/mL in both cases [44].

Norfloxacin loaded in the scaffolds was characterized by a microbicidal effect slightly higher against *P. aeruginosa* than against *S. aureus* and, moreover, scaffolds loaded with the free drug seem to have an antimicrobial activity higher than those loaded with norfloxacin in the nanocomposite. These could be due to the slower drug release of hybrid scaffolds compared to those loaded with norfloxacin as a free drug. However, the antimicrobial activity was sustained for 48 h.

Although a certain margin of error could be evidenced by the high variability of the results, a significant antimicrobial effect was achieved since all the scaffolds were able to decrease the bioburden by at least 100-fold (a two-log reduction). This suggests that upon implant, the scaffolds were effective for controlling and decreasing bacteria proliferation.

## 4. Conclusions

Scaffolds entirely based on polysaccharides (pullulan and chitosan plus chondroitin sulfate or hyaluronic acid) were manufactured by means of electrospinning and norfloxacin was loaded as a free drug or as nanocomposite of montmorillonite. The scaffolds were characterized by their homogeneous structures, with fibers of 500 nm diameter when norfloxacin was loaded as a free drug, independent of drug concentration. On the contrary, the presence of nanocomposite caused a certain degree of surface roughness of the fibers with 1000 nm diameters, dramatically influenced by drug concentration. Moreover, this altered entanglement of polymer chains in the scaffolds and caused higher deformability and lower elasticity, compared to the scaffolds loaded with norfloxacin as a free drug, and decreased the mechanical resistance of the systems. The hydration of the scaffolds changed their mechanical properties and the scaffolds were more prone to deformation. This is an advantageous feature, considering their implantation in lesions. Moreover, scaffold degradation occurring via lysozyme secreted during the inflammatory phase of the healing process should ensure scaffold resorption and, simultaneously, drug release. All the scaffolds proved to be degraded via lysozyme and this sustained the drug release (from 50% to 100% in 3 days, depending on system composition), especially when the drug was loaded in the scaffolds as a nanocomposite at 1%. Moreover, the scaffolds were able to decrease the bioburden by at least 100-fold, proving that drug loading in the scaffolds did not impair the antimicrobial activity of norfloxacin. Chondroitin sulfate and montmorillonite in the scaffolds proved to possess a synergic performance in enhancing the fibroblast proliferation without impairing norfloxacin antimicrobial properties. The scaffold based on chondroitin sulfate and containing 1% norfloxacin in nanocomposite was demonstrated to possess adequate stiffness to support fibroblast proliferation and the capability to sustain antimicrobial properties to prevent/treat nonhealing wound infection during the healing process.

## 5. Patents

Sandri, G., Bonferoni, M.C., Rossi, S., Ferrari, F., electrospun nanofibers and membranes, PCT/IT2017/000160, 2017.

## Figures and Tables

**Table 1:** Quali-quantitative composition of polymeric blends.

% *w/w*	PUL	CH	CA	CS	HA	N	VHS	H_2_O/CH_3_COOH
CH-N1	10	2.5	2.5	--	--	0.15	--	55/45
CH-N2				--	--	0.30	--	
CH-H1				--	--	0.15	0.94	
CH-H2				--	--	0.30	1.88	
CH/CS-N1				0.5	--	0.15	--	
CH/CS-N2				0.5	--	0.30	--	
CH/CS-H1				0.5	--	0.15	0.94	
CH/CS-H2				0.5	--	0.30	1.88	
CH/HA-N1				--	0.5	0.15	--	
CH/HA-N2				--	0.5	0.30	--	
CH/HA-H1				--	0.5	0.15	0.94	
CH/HA-H2				--	0.5	0.30	1.88	

## Supplementary Materials

The following are available online at https://www.mdpi.com/1999-4923/12/4/325/s1, Figure S1: FTIR spectra of all the components of the scaffolds, Figure S2: XRPD spectra of the components of the scaffolds presenting signals, Figure S3: Thermal analysis (TGA and DSC) of the components and the scaffolds containing the nanocomposite.

## References

[1] Boateng J., Matthews K., Stevens H.N., Eccleston G.M. (2008). Wound Healing Dressings and Drug Delivery Systems: A Review. J. Pharm. Sci..

[2] Velnar T., Bailey T., Smrkolj V. (2009). The Wound Healing Process: An Overview of the Cellular and Molecular Mechanisms. J. Int. Med Res..

[3] Gupta A., Mumtaz S., Li C.-H., Hussain I., Rotello V.M. (2019). Combatting antibiotic-resistant bacteria using nanomaterials. Chem. Soc. Rev..

[4] Siddiqui A.R., Bernstein J.M. (2010). Chronic wound infection: Facts and controversies. Clin. Dermatol..

[5] Percival S. (2017). Importance of biofilm formation in surgical infection. BJS.

[6] Van Giau V., An S.S.A., Hulme J. (2019). Recent advances in the treatment of pathogenic infections using antibiotics and nano-drug delivery vehicles. Drug Des. Dev. Ther..

[7] Fulaz S., Vitale S., Quinn L., Casey E. (2019). Nanoparticle-Biofilm Interactions: The Role of the EPS Matrix. Trends Microbiol..

[8] García-Villén F., Faccendini A., Aguzzi C., Cerezo P., Bonferoni M.C., Rossi S., Grisoli P., Ruggeri M., Ferrari F., Sandri G. (2019). Montmorillonite-norfloxacin nanocomposite intended for healing of infected wounds. Int. J. Nanomed..

[9] Sandri G., Rossi S., Bonferoni M.C., Miele D., Faccendini A., Del Favero E., Di Cola E., Cornaglia A.I., Boselli C., Luxbacher T. (2019). Chitosan/glycosaminoglycan scaffolds for skin reparation. Carbohydr. Polym..

[10] Sandri G., Miele D., Faccendini A., Bonferoni M.C., Rossi S., Grisoli P., Taglietti A., Ruggeri M., Bruni G., Vigani B. (2019). Chitosan/Glycosaminoglycan Scaffolds: The Role of Silver Nanoparticles to Control Microbial Infections in Wound Healing. Polymers.

[11] Sahana T.G., Rekha P.D. (2018). Biopolymers: Applications in wound healing and skin tissue engineering. Mol. Boil. Rep..

[12] Ajmal G., Bonde G.V., Thokala S., Mittal P., Khan G., Singh J., Pandey V.K., Mishra B. (2019). Ciprofloxacin HCl and quercetin functionalized electrospun nanofiber membrane: Fabrication and its evaluation in full thickness wound healing. Artif. Cells Nanomedicine Biotechnol..

[13] Nejaddehbashi F., Hashemitabar M., Bayati V., Moghimipour E., Movaffagh J., Orazizadeh M., Abbaspour M. (2019). Incorporation of Silver Sulfadiazine into An Electrospun Composite of Polycaprolactone as An Antibacterial Scaffold for Wound Healing in Rats. Cell J..

[14] Grgurić T.H., Mijović B., Zdraveva E., Bajsić E.G., Slivac I., Ujčić M., Dekaris I., Trcin M.T., Vuković A., Kuzmić S. (2020). Electrospinning of PCL/CEFUROXIM® fibrous scaffolds on 3D printed collectors. J. Text. Inst..

[15] Abdallah O., Jalali F., Zamani S., Isamil H.I., Ma S., Nasrallah G.K., Younes H.M. (2016). Fabrication an Characterization of 3D electrospun biodegradable nanofibers for wound dressing, drug delivery and other tissue engineering applications. Pharm. Nanotechnol..

[16] Mehta P., Zaman A., Smith A., Rasekh M., Haj-Ahmad R., Arshad M.S., Der Merwe S., Chang M.-W., Ahmad Z., Van Der Merwe S. (2018). Broad Scale and Structure Fabrication of Healthcare Materials for Drug and Emerging Therapies via Electrohydrodynamic Techniques. Adv. Ther..

[17] Chen S., Liu B., A Carlson M., Gombart A.F., A Reilly D., Xie J. (2017). Recent advances in electrospun nanofibers for wound healing. Nanomedicine.

[18] Kupiec T.C., Matthews P., Ahmad R. (2013). Dry-heat sterilization of parenteral oil vehicles. Int. J. Pharm. Compd..

[19] Cordenonsi L.M., Faccendini A., Rossi S., Bonferoni M.C., Malavasi L., Raffin R., Schapoval E.E.S., Del Fante C., Vigani B., Miele D. (2019). Platelet lysate loaded electrospun scaffolds: Effect of nanofiber types on wound healing. Eur. J. Pharm. Biopharm..

[20] Pharmaceutical Quality/CMC (2019). Transdermal and Topical Delivery Systems—Product Development and Quality Considerations, Guidance for Industry. U.S. Department of Health and Human Services.

[21] Mahmoud A.A., Salama A. (2016). Norfloxacin-loaded collagen/chitosan scaffolds for skin reconstruction: Preparation, evaluation and in-vivo wound healing assessment. Eur. J. Pharm. Sci..

[22] Samanidou V.F., Demetriou C.E., Papadoyannis I.N. (2003). Direct determination of four fluoroquinolones, enoxacin, norfloxacin, ofloxacin, and ciprofloxacin, in pharmaceuticals and blood serum by HPLC. Anal. Bioanal. Chem..

[23] Sandri G., Bonferoni M.C., Ferrari F., Rossi S., Aguzzi C., Mori M., Grisoli P., Cerezo P., Tenci M., Viseras C. (2014). Montmorillonite–chitosan–silver sulfadiazine nanocomposites for topical treatment of chronic skin lesions: In vitro biocompatibility, antibacterial efficacy and gap closure cell motility properties. Carbohydr. Polym..

[24] Huan S., Liu G., Han G., Cheng W., Fu Z., Wu Q., Wang Q. (2015). Effect of Experimental Parameters on Morphological, Mechanical and Hydrophobic Properties of Electrospun Polystyrene Fibers. Materials.

[25] Yu X., Zipp G.L., Iii G.W.R.D. (1994). The Effect of Temperature and pH on the Solubility of Quinolone Compounds: Estimation of Heat of Fusion. Pharm. Res..

[26] Irwin N., McCoy C.P., Carson L. (2013). Effect of pH on the in vitro susceptibility of planktonic and biofilm-grown Proteus mirabilis to the quinolone antimicrobials. J. Appl. Microbiol..

[27] Sandri G., Faccendini A., Longo M., Ruggeri M., Rossi S., Bonferoni M.C., Miele D., Prina-Mello A., Aguzzi C., Iborra C.V. (2020). Halloysite- and Montmorillonite-Loaded Scaffolds as Enhancers of Chronic Wound Healing. pharmaceutics.

[28] Rabbani M., Bathaee H., Rahimi R., Maleki A. (2016). Photocatalytic degradation of p -nitrophenol and methylene blue using Zn-TCPP/Ag doped mesoporous TiO 2 under UV and visible light irradiation. Desalin. Water Treat..

[29] Tran T., Hamid Z., Cheong K. (2018). A Review of Mechanical Properties of Scaffold in Tissue Engineering: Aloe Vera Composites. J. Physics: Conf. Ser..

[30] Pawlaczyk M., Lelonkiewicz M., Wieczorowski M. (2013). Age-dependent biomechanical properties of the skin. Adv. Dermatol. Allergol..

[31] Hadjipanayi E., Mudera V., Brown R.A. (2009). Close dependence of fibroblast proliferation on collagen scaffold matrix stiffness. J. Tissue Eng. Regen. Med..

[32] Clark R.A. (1993). Biology of Dermal Wound Repair. Dermatol. Clin..

[33] El-Mohri H., Wu Y., Mohanty S., Ghosh G. (2017). Impact of matrix stiffness on fibroblast function. Mater. Sci. Eng. C.

[34] Saporito F., Sandri G., Rossi S., Bonferoni M.C., Riva F., Malavasi L., Caramella C., Ferrari F. (2018). Freeze dried chitosan acetate dressings with glycosaminoglycans and traxenamic acid. Carbohydr. Polym..

[35] Perez H.A., Bustos A., Taranto M.P., Frias M., Ledesma A.E. (2018). Effects of Lysozyme on the Activity of Ionic of Fluoroquinolone Species. Molecules.

[36] Islam N., Wang H., Maqbool F., Ferro V. (2019). In Vitro Enzymatic Digestibility of Glutaraldehyde-Crosslinked Chitosan Nanoparticles in Lysozyme Solution and Their Applicability in Pulmonary Drug Delivery. Molecules.

[37] Dua K., Malipeddi V.R., Madan J.R., Gupta G., Chakravarthi S., Awasthi R., Kikuchi I.S., Pinto T.D.J.A. (2016). Norfloxacin and metronidazole topical formulations for effective treatment of bacterial infections and burn wounds. Interv. Med. Appl. Sci..

[38] Öztürk E., Agalar C., Öztürk E. (2004). Norfloxacin-loaded Chitosan Sponges as Wound Dressing Material. J. Biomater. Appl..

[39] Sandri G., Aguzzi C., Rossi S., Bonferoni M.C., Bruni G., Boselli C., Cornaglia A.I., Riva F., Viseras C., Caramella C. (2017). Halloysite and chitosan oligosaccharide nanocomposite for wound healing. Acta Biomater..

[40] Sandri G., Bonferoni M.C., Rossi S., Ferrari F., Aguzzi C., Iborra C.V., Caramella C., Ågren S.M. (2016). Clay minerals for tissue regeneration, repair, and engineering. Wound Healing Biomaterials.

[41] Sandri G., Bonferoni M.C., Rossi S., Ferrari F., Mori M., Cervio M., Riva F., Liakos I., Athanassiou A., Saporito F. (2014). Platelet lysate embedded scaffolds for skin regeneration. Expert Opin. Drug Deliv..

[42] Aguzzi C., Sandri G., Bonferoni M.C., Cerezo P., Rossi S., Ferrari F., Caramella C., Iborra C.V. (2014). Solid state characterisation of silver sulfadiazine loaded on montmorillonite/chitosan nanocomposite for wound healing. Colloids Surf. B Biointerfaces.

[43] Rossi S., Marciello M., Sandri G., Ferrari F., Bonferoni M.C., Papetti A., Caramella C., Dacarro C., Grisoli P. (2007). Wound Dressings Based on Chitosans and Hyaluronic Acid for the Release of Chlorhexidine Diacetate in Skin Ulcer Therapy. Pharm. Dev. Technol..

[44] Norrby S.R., Jonsson M. (1983). Antibacterial activity of norfloxacin. Antimicrob. Agents Chemother..

