# Supplementary material for: Norfloxacin-Loaded Electrospun Scaffolds: Montmorillonite Nanocomposite vs. Free Drug
Source: Pharmaceutics. 2020 Apr 4;12(4):325. doi: 10.3390/pharmaceutics12040325 (PMC7238150; doi:10.3390/pharmaceutics12040325)
Supplement: Supplementary file 1 [file pharmaceutics-12-00325-s001.pdf]

pharmaceutics

MDPI

Supplementary Materials: Norfloxacin Loaded Electrospun Scaffolds: Montmorillonite Nanocomposite vs. Free Drug

Angela Faccendini, Marco Ruggeri, Dalila Miele, Silvia Rossi, Maria Cristina Bonferoni, Carola Aguzzi, Pietro Grisoli, Cesar Viseras, Barbara Vigani, Giuseppina Sandri, Franca Ferrari

Figure S1 reports the FTIR spectra of all the components of the scaffolds.

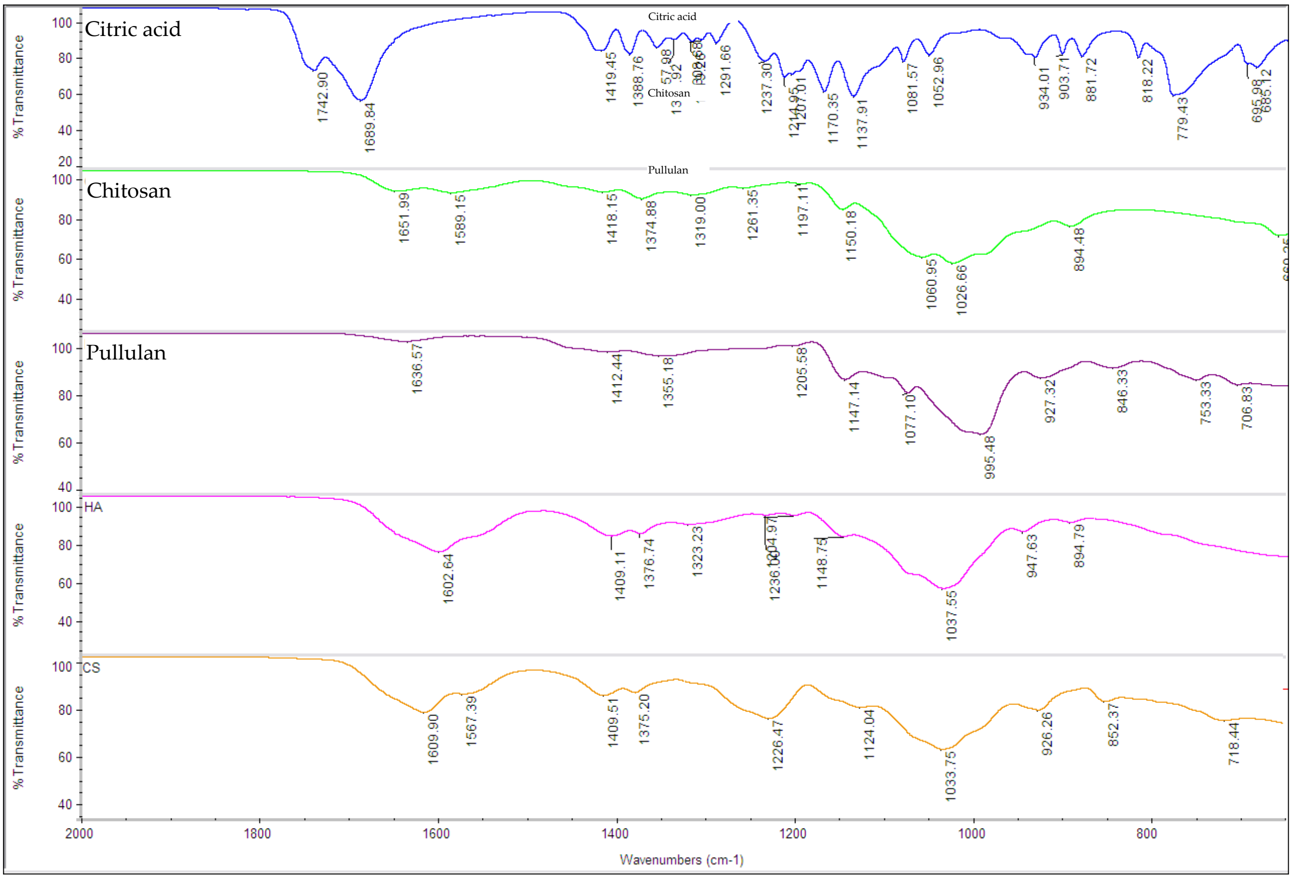

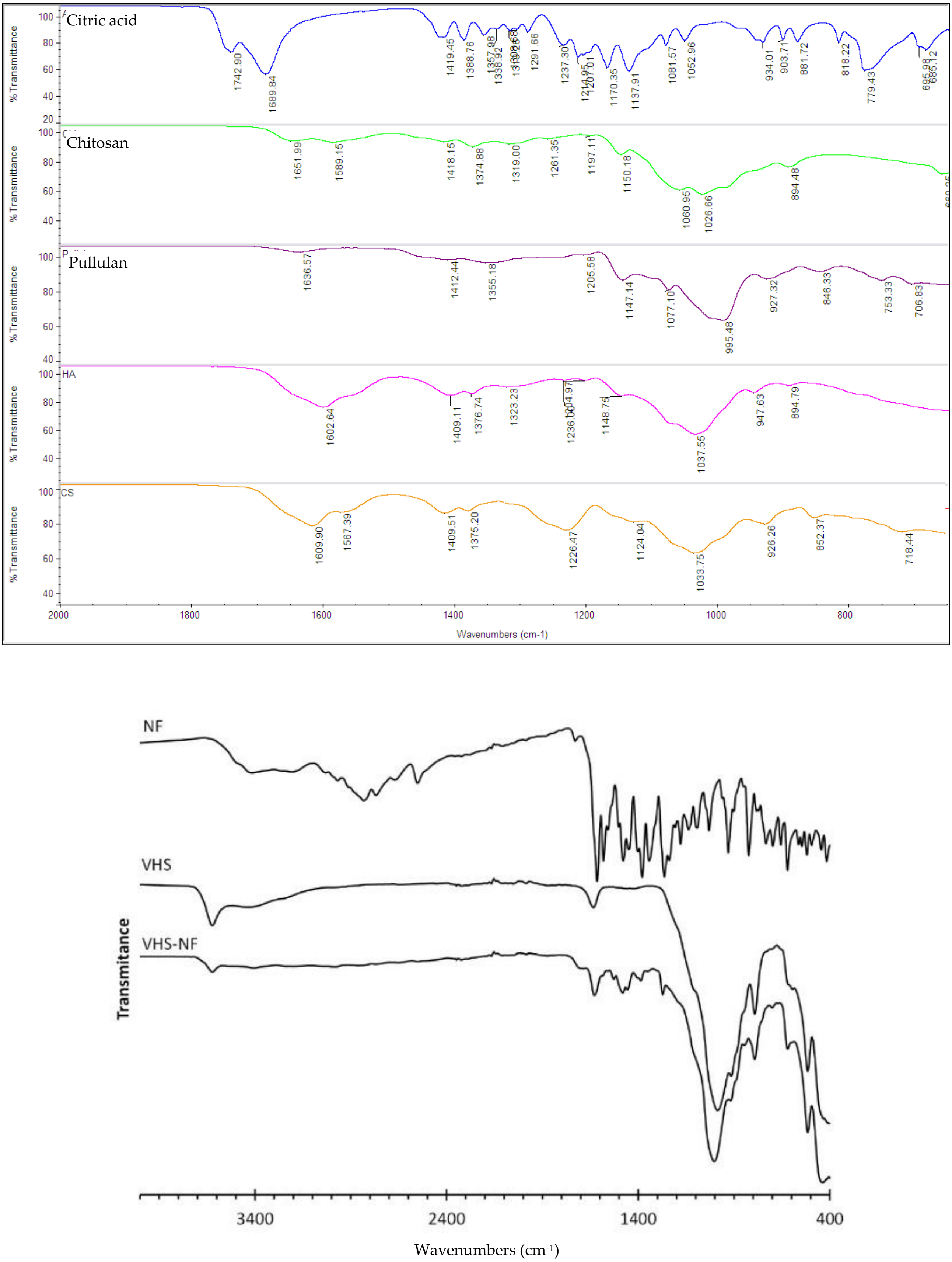

Figure S1. FTIR spectra of all the components of the scaffolds.

Figure S2 reports the XRPD spectra of the components of the scaffolds presenting signals.

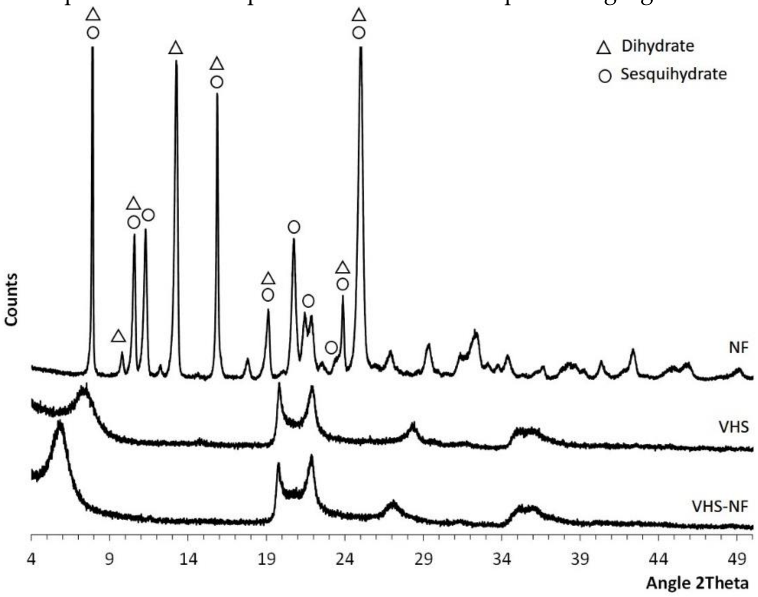

Figure S2. XRPD spectra of the components of the scaffolds presenting signals. (from García-Villén, F., Faccendini, A., Aguzzi, C., Cerezo, P., Bonferoni, M. C., Rossi, S., Grisoli, P., Ruggeri M., Ferrari, F., Sandri, G., Viseras, C. Montmorillonite-norfloxacin nanocomposite intended for healing of infected wounds. *Int J Nanomedicine*, **2019**, *14*, 5051–5060. DOI: 10.2147/IJN.S208713).

Thermal analysis

Thermogravimetric analysis (TGA) (mod. TGA-50H, Shimadzu) was performed using a vertical oven and a precision of 0.001 mg. Approximately 40 mg of each sample were weighted in aluminum sample pans. The experiments were performed in 30–950 °C range, atmospheric air and a heating rate of 10 °C/min. Additionally, differential scanning calorimetry (DSC) analyses were done in a Mettler Toledo and using aluminum crucibles. The temperature range was defined between 30° C and 400 °C at a heating rate of 10 °C/min. All the analyses were done in atmospheric air.

Figure S3 reports the thermal analysis (TGA and DSC) of the components and the scaffolds containing the nanocomposites.

The TGA analysis showed total drug decomposition before 630ºC. The first weight loss occurred from 30 °C to 196 °C, with an inflection point at 100 °C. This step is ascribable to the evaporation of water and coincided with the first endothermic event of the corresponding DSC curve. Nonetheless, the presence of an inflection point could be related to different types of crystallization water. In fact, different hydrated forms of norfloxacin has been reported over the literature such as dihydrate norfloxacin and sesquihydrate norfloxacin, among others (Katdare et al., 1986; Mazuel, 1991; Basavoju et al., 2006; Sadeek et al., 2006; Roy et al., 2008; Puigjaner et al., 2010; Rusu et al., 2016; Nunes et al., 2018). Particularly, according to Mazuel (1991), the dihydrate form of NF give rise to a DSC profile in which evaporation of water is composed by two different steps that were not visible in this case. The sharp and intense endothermic peak located at 227 °C (T onset 218 °C) corresponds with the typical norfloxacin melting point, confirmed by the absence of weight loss in the TGA curve of NF. Decomposition of the drug initiated at 300 °C according to TGA and was formed by three overlapped steps (inflection point at ~370 °C and ~500 °C). These steps have been previously attributed to the loss of different functional groups of the norfloxacin molecule ($6C_2H_2+3NO+HF+1/2H_2$) by Sadeek et al. (2006) and/or gaseous products (Nunes et al., 2018). The maximum of the exothermic event showed by DSC was located at 370 °C, coinciding with the first inflection point of the TGA.

TGA thermogram of VHS showed a first slight weight loss of 2% (*w/w*), which corresponded to low amounts of hydration water. Typical clay minerals dehydroxylation step had its onset at about 600ºC and finished at 720 °C (Földvári, 2011). This weight loss accounted for 3% (*w/w*) of VHS sample. The absence of decomposition of impurities related to montmorillonite clay minerals (such as quartz, mullite, corderite, cristobalite) (Földvári, 2011), which occurs from 800 °C onwards, confirmed the high purity of VHS. DSC thermogram generated by VHS confirmed the stability of the clay mineral in the temperature range evaluated, the low amount of water loss not being detectable.

The VHS-NF nanohybrid suffered a continuous weight loss during throughout the TGA thermogram due to overlapping events: water loss followed by the decomposition of NF and dehydroxylation of clay. Considering that NF

fully decomposed before 630 °C, differential weight loss between VHS and VHS-NF revealed the amount of drug in the nanohybrid, which accounted for ~16% (*w/w*). Moreover, it was also possible to affirm that the drug was not in its crystalline form in VHS-NF, since its fusion event was totally absent in VHS-NF DSC.

TGA and DSC curves of nanofiber-based scaffolds (membranes A to C) are included in Figure S3. The three scaffolds were characterized by a slight weight loss at the beginning of the TGA thermogram corresponding to hydration water evaporation. This water loss accounted for a ~8% *w/w* in membrane A (30–110 °C) and ~7% *w/w* in membranes B and C (30–101 °C). Although not very visible, DSC analysis confirmed this weight loss step by the presence of a slight endothermic event between 30 and 110ºC for all the scaffolds.

Additionally, for the three scaffolds, at 230 °C T onset there were peaks corresponding to a marked weight loss. CH scaffold presented ~300 °C inflection point and the weight loss event ended at 324 °C. Moreover, a further small weight loss (3% *w/w*) occurred between 324 °C and 500 °C and this seems due to the chitosan decomposition. These events were overlapped with PUL decomposition occurred at ~300 °C. DSC thermogram confirmed the presence of the overlapped TGA events with the presence of two endothermic peaks centered at 280 °C and 304 °C, respectively. It is conceivable that both chitosan and pullulan forming the scaffolds decomposed in overlapped events. The presence of HA and CS in scaffolds did not dramatically changed the thermal behavior of the scaffolds. CH/HA and CH/CS scaffolds were characterized by a weight loss starting at 230 °C (same point as CH scaffolds) and ending at 303 °C for CH/HA scaffolds and at 310 °C for CH/HA scaffolds (26% *w/w* of mass loss for both of them). A further weight loss occurred at ~406–430 °C for both the scaffolds (15% *w/w* of mass loss). Once again, both weight loss events coincided with endothermic events in DSC thermograms. DSC patter suggested a slight increase in thermal stability of CH/CS and CH/HA scaffolds compared to CH scaffolds, since all the events highlighted for CH scaffolds were present at higher temperatures probably due to the interaction between CH and both HA and CS.

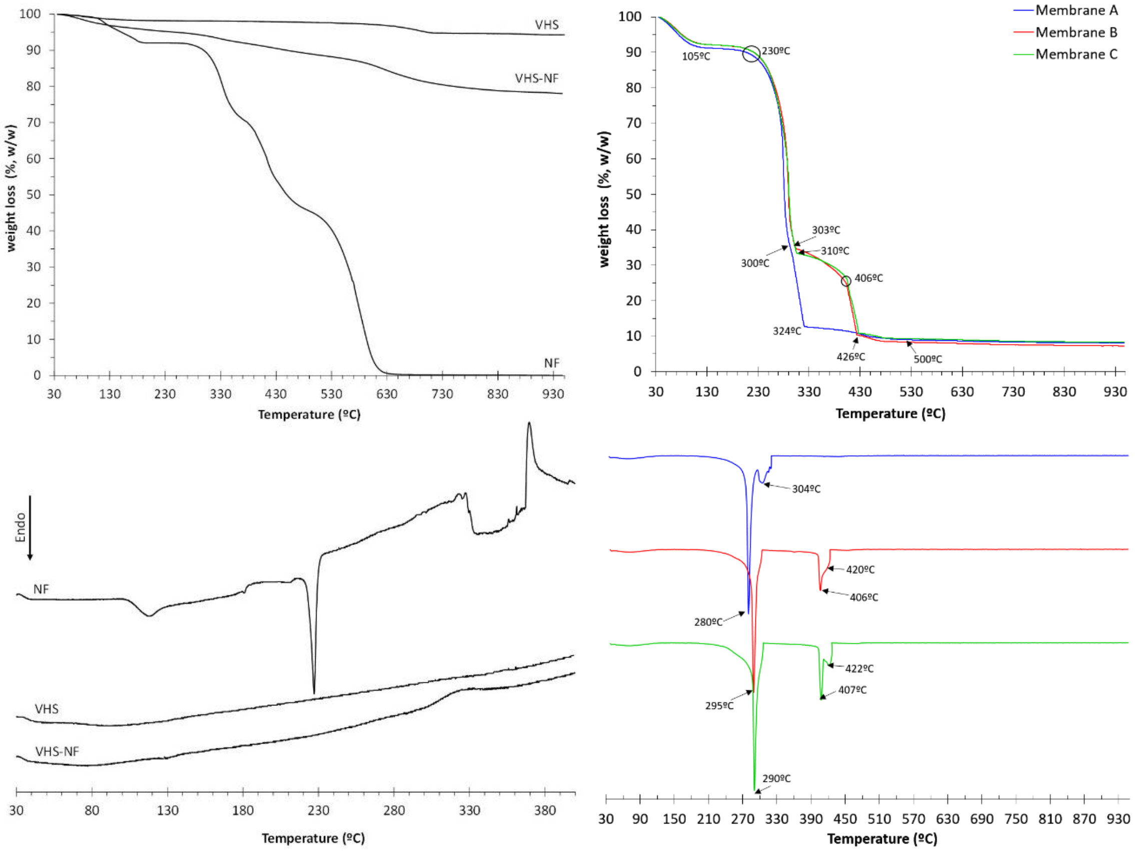

Figure S3. Thermal analysis (TGA and DSC) of the components and the scaffolds containing the nanocomposites. (from García-Villén, F., Faccendini, A., Aguzzi, C., Cerezo, P., Bonferoni, M. C., Rossi, S., Grisoli, P., Ruggeri M., Ferrari, F., Sandri, G., Viseras, C. Montmorillonite-norfloxacin nanocomposite intended for healing of infected wounds. Int J Nanomedicine, 2019, 14, 5051–5060. DOI: 10.2147/IJN.S208713)

References

Basavoju, S., Bostrom, D., and Velaga, P. (2006). Pharmaceutical cocrystals and salts of Norfloxacin. Crystal Growth and Design, 6(12), 2699–2708.

Földvári, M. (2011). Handbook of thermogravimetric system of minerals and its use in geological practice. (M. Gyula, O.P. Dezsö Simonyi, and F. Tamás, Eds.) (Vol. 213). Budapest: Innova-Print Kft.
Földvári, M. (2011). Handbook of thermogravimetric system of minerals and its use in geological practice. (M. Gyula, O.P. Dezsö Simonyi, and F. Tamás, Eds.) (Vol. 213). Budapest: Innova-Print Kft.
Katdare, A.V., Ryan, J.A., Bavitz, J.F., Erb, D.M., Guillory, J.K. (1986). Characterization of hydrates of Norfloxacin. MikrochimicaActa, III, 1-12.

Mazuel, C. (1991). Norfloxacin. In K. Florey, H. Brittain, D. Mazzo, T. Wozniak, G. Brenner, G. Forcier, and A. Al-Bad (Eds.), Analytical profiles of drug substances and excipients (1st edition, pp. 557–600). Academic Press Inc.

Puigjaner, C., Barbas, R., Portell, A., Font-Bardia, M., Alcobé, X., Prohens, R. (2010). Revisiting the solid state of norfloxacin. Crystal Growth and Design, 10, 2948-2953.

Roy, S., Rajesh Goud, N., JagadeeshBabu, N., Iqbal, J., Kruthiventi, A.K., and Nangia, A. (2008). Crystal structures of norfloxacin hydrates. Crystal Growth and Design, 8(12), 4343–4346.

Rusu, A., Hancu, G., Tóth, G., Toma, F., Mare, A.D., Man, A., Uivaroși, V. (2016). Synthesis, characterization and microbiological activity evaluation of two silver complexes with norfloxacin. Farmacia, 64(6), 922–932.
